# Supplementary material for: Thioacetamide-Induced Acute Liver Injury Increases Metformin Plasma Exposure by Downregulating Renal OCT2 and MATE1 Expression and Function
Source: Biomedicines. 2023 Dec 15;11(12):3314. doi: 10.3390/biomedicines11123314 (PMC10741527; doi:10.3390/biomedicines11123314)
Supplement: Supplementary file 1 [file biomedicines-11-03314-s001.zip › biomedicines-2728208-supplementary.pdf]

Supporting Information for

ORIGINAL ARTICLE

Table S1. Primer sequences for qPCR.

**Gene**	Forward（5'-3'）	Reverse（5'-3'）
Rat *OCT2*	ATGAAATGGTCTGCCTGGTC	ATGTCACACATGGAGGAGCA
Rat *MATE1*	CATCGGGATCTCACTGATGTTC	GATGATTCCTGACCACAGACCAA
Rat ACTIN	GCTATGTTGCCCTAGACTTCG	GCCACAGGATTCCATACCCAG
